# A Missense Mutation in the *UGDH* Gene Is Associated With Developmental Delay and Axial Hypotonia

**DOI:** 10.3389/fped.2020.00071

**Published:** 2020-02-27

**Authors:** Kheloud M. Alhamoudi, Javaid Bhat, Marwan Nashabat, Masheal Alharbi, Yusra Alyafee, Abdulaziz Asiri, Muhammad Umair, Majid Alfadhel

**Affiliations:** ^1^Medical Genomics Research Department, King Abdullah International Medical Research Center (KAIMRC), King Saud Bin Abdulaziz University for Health Sciences, King Abdulaziz Medical City, Ministry of National Guard Health Affairs, Riyadh, Saudi Arabia; ^2^Medical Core Facility and Research Platforms, King Abdullah International Medical Research Center (KAIMRC), King Saud Bin Abdulaziz University for Health Sciences, King Abdulaziz Medical City, Ministry of National Guard Health Affairs, Riyadh, Saudi Arabia; ^3^Division of Genetics, Department of Pediatrics, King Abdullah Specialized Children's Hospital, King Saud Bin Abdulaziz University for Health Sciences, King Abdulaziz Medical City, Ministry of National Guard Health Affairs, Riyadh, Saudi Arabia

**Keywords:** UGDH, hypotonia, GDD, missense, Saudi population, rare genetic disease

## Abstract

UDP-glucose dehydrogenase (*UGDH*) encodes an oxidoreductase that converts two successive oxidations of UDP-glucose to produce UDP-glucuronic acid, a key component in the synthesis of several polysaccharides such as glycosaminoglycan and the disaccharide hyaluronic acid. UGDH is critical to the production of extracellular matrix components which are essential to the migration and connectivity of neurons early in human brain development. In this report, we describe one child of a consanguineous family who presented with distinct clinical features including global developmental delay, axial hypotonia, bilateral undescended testis, and subtle dysmorphic features. Whole genome sequencing and a segregation was performed to identify the genetic cause of the disease within the family. Though mutations in the UGDH protein have been described as causing developmental delay in various model organisms, to our knowledge, this is the first identification of the novel homozygous missense variant in exon8 of *UGDH* NM_003359.3: c.950 G>A (p.Arg317Gln) and most likely the cause of the patient's phenotype. This variant falls in an active region and replaces the highly conserved Arginine 317 residues across mammals.

## Introduction

Neurological and neurodevelopmental disorders represent the largest category of Mendelian genetic inheritance diseases in humans. They include a variety of clinical features and characterizations that range from common to very rare clinical presentations (1, 2). Therefore, these variances are considered a major obstacle to discerning and assigning the molecular classifications of these disorders. However, a broad spectrum of genomics applications such as karyotyping and comparative genome hybridization array (CGH) (3) have been tremendously advantageous in genomic studies, particularly in unraveling the etiology of genetic causes in patients suspected to have a Mendelian disease (4–6). Indeed, the advances in recent genomic next-generation sequencing technologies such as whole exome sequencing (WES) and whole genome sequencing (WGS) have significantly enabled the identification of *de novo*, inherited, rare, and novel disease-associated variants in patients with genetically heterogeneous conditions, including all neurological diseases such as developmental abnormalities (6, 7). Although the rate of discoveries of novel genes and variants that contribute to common neurological diseases, including Parkinson's disease and Alzheimer's disease, is continually growing (8–10), only a few genetic risk factors for rare neurological disease have been identified; the majority remain largely unknown.

**Figure 1:**
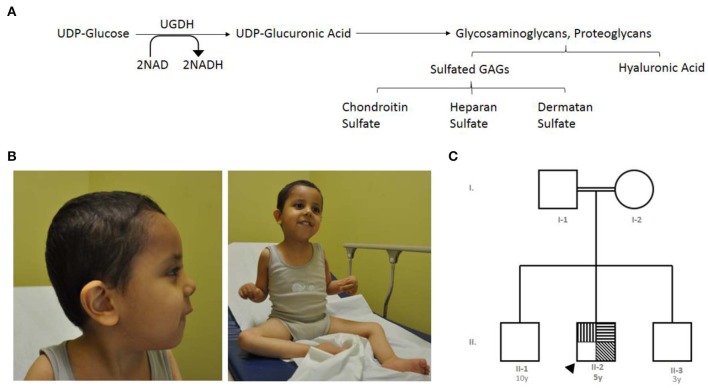
The index's case presentation and genetics pedigree information. **(A)** Metabolic mechanism of UDP-glucose dehydrogenase catalysis, UGDH catalyzes two successive oxidations of the UDP-glucose and converts it to UDP-glucuronate, UGDH convert of nicotinamide adenine dinucleotide (NAD) to produce the cofactor nicotinamide adenine dinucleotide phosphate (NADH). UDP-glucuronic acid is an essential sugar nucleotide precursor in the synthesis of polysaccharides, such as Sulfated GAGs and hyaluronic acid. **(B)** Clinical features of the index with global developmental delay, axial hypotonia, bilateral undescended testis, and subtle dysmorphic features. **(C)** Family pedigree constructed from the details provided by the index's mother, generated using (https://www.progenygenetics.com), squares (males); circles (females); annotated symbols (affected individuals); open symbols (unaffected individuals); arrowheads (Index). 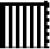 Axial hypotonia; 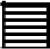 Global developmental delay; 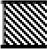 dysmorphic features.

The *UGDH* gene, officially known as UDP-glucose dehydrogenase, is a gene found in chromosome 4q15.1 (11). It encodes an oxidoreductase that catalyzes two successive oxidations of the UDP-glucose (UDP-Glc) and converts it to UDP-glucuronic acid (UDP-GlcA), concurrent with orchestrating the conversion of nicotinamide adenine dinucleotide (NAD) to produce the cofactor nicotinamide adenine dinucleotide phosphate (NADH) (11–13). UDP-GlcA is an essential sugar nucleotide precursor and a key component in the synthesis and stepwise degradation of glycosaminoglycans (GAGs). GAGs are constitute either as an unsulfated anionic linear polysaccharides composed of repeating disaccharide units, such as hyaluronic acid (glucuronic acid and N-acetylglucosamine) (14) or as several sulfated polysaccharides such as Chondroitin sulfate (CS), dermatan sulfates (DS), heparan sulfate (HS) and Keratan sulfate (KS) (15) (Figure 1A). Polymeric GAGs have the ability to either covalently bind through a linkage region to core proteins to produce proteoglycans (PGs) (16, 17) or remain as free polysaccharides in the extracellular matrix (ECM) found in multiple connective tissues. These GAGs and PGs fulfill essential roles in diverse biological cellular processes, such as the migration, differentiation and connectivity of neurons early in human brain development (18, 19). Defects in genes that are associated with glycosylation cause many rare genetic disorders presenting phenotypes such s global developmental delay, hypotonia, intellectual disability, metabolic defects and other defects (20). The cellular functions of *UGDH* have been studied extensively in various model organisms, including *Caenorhabditis elegans, Drosophila melanogaster*, and zebrafish (21–23). Such studies have been conducted using targeted gene disruption; collectively, they all demonstrated the importance of *UGDH* in cellular signaling, developmental processes and cardiac valve formation (23–25).

In this study, we report the identification of a novel missense genetic substitution, which is predicted to be pathogenic, in exon 8 of *UGDH* (c.950G>A; p.Arg317Gln) in one patient with global developmental delay, axial hypotonia, bilateral undescended testis, and subtle dysmorphic features, via the application of WGS technology.

## Materials and Methods

### Human Subjects

The index underwent a full routine clinical evaluation, including dysmorphological examinations, neurological, radiological and several genetic evaluations such as molecular karyotyping, WES, and WGS was conducted at king Abdulaziz Medical City in Riyadh, Saudi Arabia. Ethical approval of this study was obtained from King Abdullah International Medical Center Institutional Review Board (IRB) study number# RC18/017/R. The standard informed clinical consent form was obtained and signed from the index's parents. The study was conducted in accordance with the tenets of the Declaration of Helsinki. Additionally, written informed consent was obtained from the parents of the patient for the publication.

### Whole Genome Sequencing and Bioinformatic Analysis

Blood samples were collected from the affected index, his parents and siblings in EDTA tubes for DNA extraction. Genomic DNA (gDNA) was isolated using QIAamp Blood midi kit (Qiagen). The target sequences of the gDNA samples were fragmented by sonication and Illumina adapters were ligated to generated fragments for subsequent sequencing on the HiSeqX platform (Illumina Inc., San Diego, CA, USA) to yield an average coverage depth of ~30X. A trio of whole exome and whole genome sequencing analyses (of the proband and both parents) was performed at Centogene a certified clinical diagnostic laboratory (Rostock, Germany).

### Variant Filtration Steps

A bioinformatics pipeline including base calling, primary filtering of low-quality reads, and probable artifacts, as well as annotation of variants, was performed by Centogene (https://www.centogene.com/). Briefly, copy number variation (CNV) calling based on the HAS pipeline was used. Following this, all disease-causing variants reported in the Human Gene Mutation Database (26), ClinVar (27), or in the latest database available from CentoMD®, as well as all variants with a minor allele frequency of <1% in the Exome Aggregation Consortium database (ExAC) (28) were considered. The general assertion criteria for variant classification are available to the public on the GeneDx ClinVar submission page (http://www.ncbi.nlm.nih.gov/clinvar/submitters/26957/).

The evaluations were focused on coding exons along with flanking +/– 20 intronic bases; however, they were extended to the complete gene region for candidate genes or in the search for a second previously described variant in the autosomal recessive inheritance pattern. All pertinent inheritance patterns were considered. In addition, the family history and clinical information provided were used to evaluate the variants that were eventually identified. All identified variants were evaluated with respect to their pathogenicity and causality. All variants related to the phenotype of the patient, except for benign or likely benign variants, were reported. CNVs of unknown significance were not reported.

### Sanger Sequencing

Sanger sequencing was employed to validate the presence of c.950G>A variant seen in WGS as well as the segregation of the identified variant within the family member along with an independent normal sample used as a negative control. Given the position of the variant, primers were designed to flank and amplify the 950G>A in exon 8 of the *UGDH* gene. UGDH_forward, 5- TGTCATAGGCTGTGCCTCTTT-3; UGDH_Reverse, 5-TTTGAATGCAAATCCCAAAA-3. And a standard sequencing reaction was performed using BigDye Terminator following the manufacturer's instruction and annealing temperature of 58°C.

### Protein Modeling of UGDH

The human UGDH structure (PDB ID 2Q3E) retrieved from the RCSB Protein Data Bank (PDB) (29) was manually mutated in the PyMOL program (https://pymol.org) to generate a model for human UGDH R317Q. Protein sequences were retrieved from the UniProt database and aligned using UniProt Align (https://www.uniprot.org/align/). Final alignment with the secondary structural elements mapped was generated in ESPript (http://espript.ibcp.fr) (30).

## Results

### Phenotypic Presentation

The index case is a 5 year-old boy, whose mother experienced an uneventful pregnancy and a spontaneous vaginal delivery. Immediately following birth, he was admitted to the nursery for 2 days due to neonatal jaundice and was then discharged in good health. At the age of 2 months, the mother noticed that he was weak and could not support his head (Figure 1B).

The patient was referred to the genetics team for the first time at the age of 13 months due to delayed developmental skills, global joint hyperlaxity, and axial hypotonia. At that time, the patient was not able to sit or support his head; he was able to say only one word and had a social smile. No seizures or concerns about his vision or hearing were noted. A physical examination revealed that all his growth parameters were below the third percentile (length, 87 cm; weight, 7.2 kg; and head circumference, 43.5 cm). Subtle dysmorphic features, including bifrontal narrowing, bulbous nose, smooth philtrum, and high arched plate, were also noted.

The patient is currently 5 years old and able to sit without support. He can produce sounds and can laugh loudly, still says only a single word. His interaction with others has improved and understand and obey simple commands. The most recent examination revealed that his growth parameters are still below the third percentile. He has axial hypotonia with some weakness in the muscles (upper limb 3/5 and lower limbs 2/5), although there has been a mild increase in his deep tendon reflexes. Radiological investigations, including brain magnetic resonance imaging and skeletal survey, were unremarkable.

### Clinical Evaluation

The index is from parents who are first cousins, consanguineous, and asymptomatic, and they have another two sons who are reportedly healthy. Figure 1C is an analysis of the pedigree constructed from the index's parents' recollections. A summary of relevant phenotypic information in two affected individuals showed that there is one paternal cousin diagnosed with sickle cell anemia and hypothyroidism, and another paternal cousin with brain atrophy. A maternal cousin was also diagnosed with an atrial septal defect. The pattern of inheritance for the remainder of the family was unremarkable. In brief, clinical and genetic information for this trait were lacking (rare) in the family members and skipped the crisscross inheritance. Thus, the observed inheritance in the proband is considered to be an autosomal recessive disorder.

### Genetic Evaluation

The patient underwent an extensive genetic and metabolic workup including karyotype, fluorescence *in situ* hybridization for Prader Willi syndrome, and basic metabolic workup; the results were unremarkable, including tandem mass spectrometry, very long chain fatty acids, urine for organic acids, and plasma amino acids. An array CGH revealed a maternal duplication of unknown clinical significance at least 1 Mb within the cytogenetic band on chromosome 8p23.1. This duplication was also detected in the unaffected mother, so it is probably not relevant. The array CGH showed a significant absence of heterozygosity, so targeted exome sequencing for the homozygosity blocks was done but the result was negative.

### Identification of the Causal Variant in *UGDH* Gene

To identify the causative variant contributing to the index's phenotype, the proband underwent clinical WES, which was initially performed through the original clinical laboratory at Centogene. However, the result was initially negative, although an overall coverage of 100% was achieved.

### Whole Genome Sequencing

**Figure 2:**
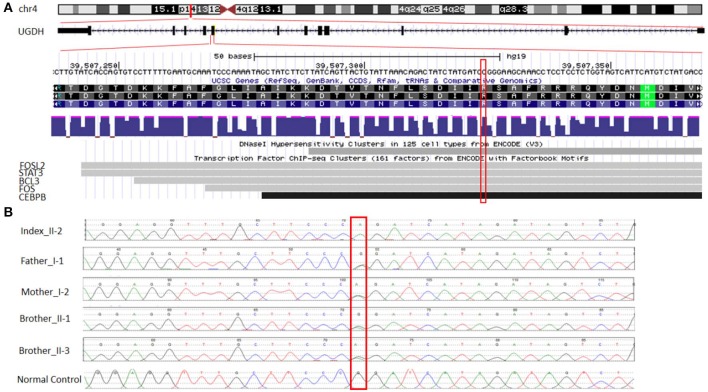
Functional annotation of the identified novel homozygous missense variant in *UGDH*. **(A)** The chromosomal location of *UGDH* NM_003359.3 and the position of the identified variant c.950 G>A. Sites of DNaseI hypersensitivity, CHIP-seq defined transcription factor binding regions from multiple independent cell lines; the darkness of the segment is proportional to the signal strength. **(B)** Segregation analysis containing the phenotype-causing missense c.950 G>A mutation in *UGDH* for index, index's father (I-1), mother (I-2), both siblings (II-1 and II-3) and a negative control sample.

A trio-WGS was requested when the patient approached his fourth year of age. A minimum of 40X coverage and a high consistency among the experiments was shown. The WGS analysis identified only a single novel homozygous missense variant in exon 8 of the *UGDH* gene Chr4 (GRCH37): g.39507325: NM_003359.3: c.950 G > A (p.Arg317Gln), which causes an amino acid change from Arg to Gln at position 317 (Figure 2A). The patient's unaffected siblings were evaluated using segregation analysis for the same variant in *UGDH* by Sanger sequencing. The segregation analysis revealed that the patient's mother, father and both his siblings were heterozygous for the same *UGDH* variant (Figure 2B).

The variant calling analysis, including the pathogenicity analysis for this candidate missense variant, was determined based on various *in silico* parameters, including the pathogenic scores obtained using SIFT (31) (deleterious), PolyPhen (32) (probably damaging), MutationTaster (33) (“disease-causing”), and conservation (nt high/aa high). Initial codon variants and filtered missense variants were finally evaluated according to the recommendations of Centogene, the American College of Medical Genetics and Genomics, and the Association for Molecular Pathology's standards and guidelines (34), ENCODE annotation on the UCSC genome browser revealed that the variant falls in a functional region and thus predicted to be pathogenic (Figure 2A). The identified variant was not observed in the homozygous state in the ExAC or genomAD and in-house 2,000 exome database.

### *In silico* Structural Analysis of the R319Q Variant of UGDH

**Figure 3:**
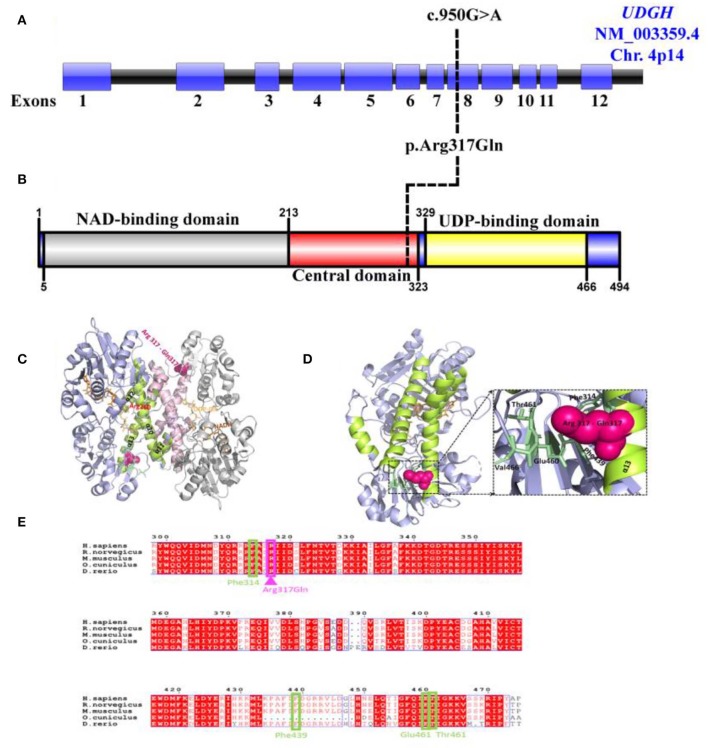
Predicted effect of R317Q mutation on human UGDH structure and function. **(A)** Schematic representation of the *UGDH* (NM_003359.5) genomic region; exons (Blue), intron (Black). Black line represents the identified c.950G>A site mutation. **(B)** Schematic diagram of the identified variant p.Arg317Gln located in the central domain of UGDH protein structure. **(C)** Cartoon representation of the dimeric structure of NADH and UDP-Glc-bound hUGDH in blue (Chain E) and gray (Chain F) (PDB ID: 2Q3E), with an R317Q mutation shown in pink. Highlighted are the helices α10–α13 (lemon and light pink colors for Chains E and F, respectively) forming the inter-dimer interface. Arg260 interacting with UDP-Glc is highlighted in red. **(D)** Cartoon representation of the mimeric structure of hUGDH bound to NADH and UDP-Glc (Chain E). Highlighted are the residues (Glu460, Thr461, Phe314, and Phe439) in pale green that interact with Arg317 in the wild-type protein and may likely become lost in an R317Q mutant. **(E)** Alignment of the sequences of *H. sapiens* UGDH (UniProt: O60701) with *M. musculus* (UniProt: O70475), R. norvegicus (UniProt: 070199), *D. rerio* (UniProt: A8WGP7), and *O. cuniculus* (UniProt: G1SP48). Arg317 (in pink) and its interacting residues Glu460, Thr461, Phe314, and Phe439 (in pale green) are highlighted. The alignment file was generated using UniProt Align (https://www.uniprot.org/align/), and final processing was performed in ESPript (http://espript.ibcp.fr).

To predict how the *UGDH* variant might influence protein structure, structural coordinates of the human UGDH (hUGDH, PDB ID: 2Q3E) was used and a mutant version with Arginine 317 (Arg317) changed to Glutamine 317 (Gln317 and named as hUGDH R317Q) was generated *in-silico* by using PyMol. Human UGDH functions as a homo hexameric complex of ~330 kDa formed by trimer of dimers and stabilized by two types of interfacial interactions. The strong intra-dimeric interface is formed by interdomain helix (α10) and the C-terminal three-helical bundle (α11–α13) and the relatively weaker inter-dimeric interface is formed by the loops between β4–α5 and α13–β9 (35). Arg260 in the loop connecting helices α11and α12 forms hydrogen bonds with UDP-Glc in the active site (Figure 3A) and serves as a sensor during subunit-subunit dimer formation for the binding of UDP-Glc and a link between catalysis and role of oligomerisation in hUGDH function (35) (Figure 3B).

Arg317 locates to α13 (294–321) of the inter-subunit dimerization-associated three-helical bundle (α11–α13) of the hUGDH and is conserved across the homologs (Figure 3C). Analysis of the hUGDH, (PDB ID: 2Q3E) structure by Amino Acid Interaction (INTAA) web server (36) allowed us to calculate the Arg317 Interaction Energy Matrix (IEM) in the protein (36). The IEM analysis highlighted that Arg317 forms multiple contacts with the residues in the neighboring structural elements with strongest interactions being formed with Glu460, Thr461, Phe314, and Phe439 (Figure 3D). Importantly, Glu460, Thr461, and Phe314 are highly conserved residues across mammals and higher eukaryotes (Figure 3E) Collectively, our identified variant Arg317Gln located in highly conserved position is rare and may contribute to the stability of UGDH's core structure and thus strengthen the pathogenicity of the identified variant.

## Discussion

In this report, a novel variant in the *UGDH* gene associated with global developmental delay, axial hypotonia, bilateral undescended testis, and subtle dysmorphic features was identified. WGS analysis revealed the identification of a novel homozygous missense variant in exon 8 of *UGDH* NM_003359.3: c.950 G > A (p.Arg317Gln) for the first time.

Though number of studies have been conducted using complete gene knockout in different models including Drosophila, zebra fish and mouse, and collectively demonstrated the importance of *UGDH* in cellular FGF signaling developmental processes and cardiac valve formation. Yet, it is it is not enough described in diversifying human pathology (14, 17, 24, 37, 38). The prediction of the identified variant's functional effect may possibly explain the presented patient and would likely pose two major consequences for hUGDH. Firstly, it has been speculated that the replacement of the negatively charged and highly conserved Arg317 with the positively charged Gln317 (Arg317Gln) would lead to the loss of multiple residue to residue interactions with the positively charged residues in the neighboring structural elements, including Glu460, Thr461, Phe314, and Phe439 emanating from Arg317. Therefore, our novel variant may perhaps lead to the UGDH core structure's disability. The importance of dimer-hexamer dynamics has been previously demonstrated as functionally essential for UGDH, and shifting this equilibrium toward obligate dimer or hexamer states leads to many fold decreases in enzyme activity (decreased kcat) as well as a significant decrease in the hyaluronate production in HEK 293 cells (39). Moreover, it has been reported that two hUGDH mutations have direct effect on the enzyme quaternary structure dynamics and stability (39–41). Secondly, our results reveal that Arg317 locates at α13 (294–321) of the inter-subunit's dimerization-associated three-helical bundle (α11–α13) of the hUGDH. It has been demonstrated that the strong intra-dimeric interface is formed by an interdomain helix (α10) and the C-terminal three-helical bundle (α11–α13), while the relatively weaker inter-dimeric interface is formed by the loops between β4–α5 and α13–β9 (35). Destabilization of the helix α13 due to the replacement of Arg317 to Gln causes a change from a residue with high-to-low α-helix-forming propensity (42). Collectively, the effect would be the destabilization of the individual subunit or the whole dimer, which in turn may disturb both the dimer to hexamer equilibrium and the overall catalysis.

Giving the catalytic mechanism of UGDH as an oxidoreductase that converts UDP-Glc to UDP-GlcA. It can be speculated that the deficiency of the UGDH enzyme will defect the synthesis of its major products such as UDP-GlcA, which might defect the downstream GAGs and PGs synthesis or accumulation. A number of studies have revealed evidence regarding the importance of the GlcA product in diversifying several human physiology, development, and cellular processes (14, 17, 24, 37, 38). Similarly, Defects in HS and CS synthesis have been associated with developmental delay and intellectual disabilities (43, 44). Additionally, Mutations resulting in the defects in GAG synthesis, degradation and modifications might be associated with series of neurodegenerative diseases. These include homozygous mutations in the *EXTL3, NDST1*, and *CHSY*1 that causes developmental delay, epilepsy and intellectual disabilities (43, 45–47). Mutations that disrupt the UGDH gene have been shown to block the GAG degradation and subsequently cause undegraded GAGs and PGs to accumulate (excess) with a specific sulfated or carbohydrate residue in multiple tissues, thus leading to severe dysfunction represented by a variety of clinical features including skeletal dysplasia, mental retardation, heart valve disease, and MPS (19, 21, 22, 25, 37, 40, 48, 49). This supports the evidence that the homozygous mutation in the *UGDH* might cause GDD, hypotonia and related neurological abnormalities. Additionally, it was reported that UGDH polymorphisms were associated with epileptic in Native American population (50). Recently, several germline recessive mutations in the *UGDH* were associated with developmental epileptic encephalopathies, developmental delay and hypotonia (Table 1) (51). Further, research using animal models will be crucial for understanding the exact mechanism of UGDH underlying developmental delay.

**Table 1:** UGDH germline mutations reported to-date.

**S.no**.	**UGDH mutations**	**Protein change**	**Exon**	**Domain**	**Main phenotype**
1	c.41A>G	p.Ty14Cys	2	NAD-binding domain	IDEE
2	c.70G>A	p.Ala24Thr	2	NAD-binding domain	IDEE
3	c.125T>C	p.lle42Thr	2	NAD-binding domain	IDEE
4	c.131C>T	p.Ala44Val	2	NAD-binding domain	IDEE
5	c.193C>T	p.Arg65*	3	NAD-binding domain	IDEE
6	c.214T>G	p.Ser72Pro	3	NAD-binding domain	IDEE
7	c.244C>A	p.Ala82Thr	3	NAD-binding domain	ID. MD
8	c.347T>C	p.lle116Thr	4	NAD-binding domain	IDEE
9	c.463C>T	p.Gln155*	4	NAD-binding domain	IDEE
10	c.523C>G	p.Pro175Ala	5	NAD-binding domain	IDEE
11	c.651G>T	p.Glu217Asp	5	Central domain	IDEE
12	c.764T>C	p.lle255Thr	6	Central domain	IDEE, multiple congenital anomalies
13	c.811G>C	p.Gln271Arg	6	Central domain	IDEE, multiple congenital anomalies
14	c.907G>A	p.Val303lle	8	Central domain	NDEE
15	c.916A>G	p.Met306Val	8	Central domain	IDEE
16	c.950G>A	p.Arg317Gln	8	Central domain	IDEE
17	c.1068T>G	p.Try356*	9	UDP-binding domain	IDEE
18	c.1100A>G	p.Tyr367Cys	9	UDP-binding domain	NDEE
19	c.1177C>T	p.Arg393Trp	10	UDP-binding domain	IDEE
20	c.1228G>T	p.Ala410Ser	10	UDP-binding domain	IDEE
21	c.1324C>T	p.Arg442Trp	11	UDP-binding domain	IDEE
22	c.1328G>A	p.Arg443His	11	UDP-binding domain	NDEE
23	c.1346AA>G	p.His449Arg	11	UDP-binding domain	IDEE

IDEE, Infantile developmental epileptic encephalopathy; NDEE, Neonatal onset developmental epileptic encephalopathy; MD, Motor disorder; ID, Intellectual disability. ^*^indicates a stop codon.

In summary, our analysis identifies a single case carrying a homozygous missense variant in *UGDH* associated with the disease prevalence that includes global developmental delay, hypotonia, subtle dysmorphic features, joint laxity, and hypotonia. However, this variant has been identified in a single family, and thus, further research investigation and additional functional characterization are needed to elucidate the effect of the novel mutation Arg317Gln on the structure and function of hUGDH should be understood in detail.

## Data Availability Statement

The raw data supporting the conclusions of this article will be made available by the authors, without undue reservation, to any qualified researcher.

## Ethics Statement

The studies involving human participants were reviewed and approved by King Abdullah International Medical Research Center institutional review board. Written informed consent to participate in this study was provided by the participants and the participants' legal guardian/next of kin. Written informed consent was obtained from the individual(s), and minor(s)' legal guardian/next of kin, for the publication of any potentially identifiable images or data included in this article.

## Author Contributions

KA performed the majority of work associated with preparing, analyzing, writing, intellectual discussion, and submitting the manuscript. JB Acquired and analyzed the protein modeling, contributed to the intellectual discussion and edited the manuscript and final figures. MN summarized and reported the clinical diagnosis, management of the patient, and edited the manuscript. MAlh performed laboratory work associated with DNA extraction. YA performed work associated with Sanger sequencing and segregation analysis. MU submitting the manuscript. AA edited the manuscript. MAlf contributed to the clinical diagnosis, management of the patient and edited the manuscript. All authors read and approved the final manuscript.

### Conflict of Interest

The authors declare that the research was conducted in the absence of any commercial or financial relationships that could be construed as a potential conflict of interest.
